# Mechanisms of Bushen Tiaoxue Granules against controlled ovarian hyperstimulation-induced abnormal morphology of endometrium based on network pharmacology

**DOI:** 10.1186/s13048-023-01339-3

**Published:** 2024-01-26

**Authors:** Jia-Cheng Zhang, Hao-Lin Zhang, Xi-Yan Xin, Yu-Tian Zhu, Xin Mao, Hang-Qi Hu, Yu-Xin Jin, Rui-Wen Fan, Xiao-Hui Zhang, Yang Ye, Dong Li

**Affiliations:** 1 Department of Traditional Chinese Medicine, Peking University Third Hospital, Beijing, China; 2 Department of Radiology, Peking University Third Hospital, Beijing, China

**Keywords:** Bushen Tiaoxue Granules, Controlled ovarian hyperstimulation, Endometrium, Experimental validation, Morphology, Network pharmacology

## Abstract

Bushen Tiaoxue Granules (BTG) is an empirical Chinese herbal formula that has been used for the treatment of subfertility. The protective effect of BTG on controlled ovarian hyperstimulation (COH)-induced impaired endometrial receptivity has been reported in our previous study. This study aims to explore the mechanisms of BTG on ameliorating abnormal morphology of endometrium based on network pharmacology. Active compounds of BTG were identified via the traditional Chinese medicine systems pharmacology and UPLC-MS technology. The SwissTargetPrediction platform and HERB database were used to screen out the putative targets of BTG. Potential targets of endometrial dysfunction caused by COH were obtained from three GEO databases. Through the STRING database, the protein–protein interaction was carried out according to the cross-common targets of diseases and drugs. GO terms and KEGG pathways enrichment analyses were conducted via the Metascape database. AutoDock Vina was used for docking validation of the affinity between active compounds and potential targets. Finally, in vivo experiments were used to verify the potential mechanisms derived from network pharmacology study. A total of 141 effective ingredients were obtained from TCMSP and nine of which were verified in UPLC-MS. Six genes were selected through the intersection of 534 disease related genes and 165 drug potential targets. Enrichment analyses showed that BTG might reverse endometrial dysfunction by regulating adherens junction and arachidonic acid metabolism. Hematoxylin–eosin staining revealed that BTG ameliorated the loose and edematous status of endometrial epithelium caused by COH. The protein expression of FOXO1A, β-Catenin and COX-2 was decreased in the COH group, and was up-regulated by BTG. BTG significantly alleviates the edema of endometrial epithelium caused by COH. The mechanisms may be related to adheren junctions and activation of arachidonic acid metabolism. The potential active compounds quercetin, taxifolin, kaempferol, eriodictyol, and isorhamnetin identified from the BTG exhibit marginal cytotoxicity. Both high and low concentrations of kaempferol, eriodictyol, and taxifolin are capable of effectively ameliorating impaired hESC cellular activity.

**Supplementary Information:**

The online version contains supplementary material available at 10.1186/s13048-023-01339-3.

## Introduction

As the main intervention of assisted reproductive technology, in vitro fertilization and embryo transfer (IVF-ET) has been chosen by an increasing number of infertile couples. A systematic review demonstrated that cumulative live birth rate of IVF-ET was between 56 and 65%, and the main cause of failure was ovarian hyperstimulation syndrome (OHSS) caused by controlled ovarian hyperstimulation (COH) in IVF period [1, 2]. COH affects the reproductive outcomes mainly through the production of excessive gonadotropins to interfere with the process of embryo implantation, including endogenous hormone levels, fertilized egg quality, and endometrial receptivity [2]. It has been revealed that endometrium is only receptive in a narrow window. In humans, endometrial receptivity achieved maximum 7–10 days after ovulation [3], whereas in rat, it occurs approximately four days post coitus [4]. During blastocyst implantation, the morphology and function of endometrium change dynamically. After the trophoblast cells adhere to the luminal epithelium, tight junction of epithelial cells at the attachment sites are strengthened, with the important role of maintaining the embryo. Subsequently, the cell migration ability is enhanced and tight junction proteins are decreased, which is likely related to facilitate the invasion of the blastocyst into the endometrial stromal layer [5]. At this meanwhile, the stromal cells undergo decidualization for the early development of the embryo [6]. Although COH is a significant method to obtain high-quality and more follicles, it leads to the synchronous imbalance between the establishment of endometrial receptivity and the window of implantation, which needs emphasis.

Many efforts have been carried out to find treatments to ameliorate endometrial status. However, up to data, only hysteroscopy and hydrosalpinx treatment have been corroborated to be significantly effective in improving fertility outcomes and included in the standard care [7]. Endometrial injury is supposed to induce sterile inflammation, regulate the immune status of endometrium, and promote decidualization [8]. Considering that reduced endometrial blood flow can adversely affect pregnancy and live birth outcomes [9], some drugs that can increase uterine artery blood flow or dilate blood vessels are applied in clinics, such as aspirin, heparin, and vitamin E [10, 11]. There were also studies demonstrating that Intrauterine administration, including human chorionic gonadotropin (HCG), peripheral blood mononuclear cells, and platelet rich plasma, can improve pregnancy rate and live birth rate by inducing endometrial differentiation and implantation window, but most of these studies showed no significant difference [12, 13]. It is found that the current treatments seem to focus on changing a certain morphology or function of the endometrium, but fail to regulate the endometrial environment as a whole. A multi-target and multi-pathway strategy to improve endometrial receptivity is worth seeking.

In the field of gynecological and reproductive related diseases, such as polycystic ovarian syndrome and endometriosis, traditional Chinese medicine (TCM) is being paid more and more attention due to its efficacy [14, 15]. Bushen Tiaoxue Granules (BTG) is a Chinese herbal compound containing 12 herbs, which has been proven beneficial in clinical and animal experiments [16, 17]. In the theory of TCM, gynecological diseases are blood oriented. Due to the menstrual cycle and physiological conditions, blood deficiency is a major feature of women’s diseases. At the meanwhile, the kidney stores “essence”, which is the key for women to become pregnant and nourish the fetus. The lack of “essence” will lead to the failure of uterine maturity. Therefore, tonifying the kidney and regulating blood flow is a major principle of TCM in the treatment of gynecological infertility related diseases, and BTG follows this principle to contain herbs. In a randomized controlled trial of 156 people, 52 patients receiving frozen-thawed embryo transfer were given BTG [18]. After 3 months of treatment, the uterine blood flow distribution in the BTG group was better than that in the control group. Our previous experimental study revealed that BTG can improve the ability of endometrial angiogenesis, which is attributable to the upregulation of estrogen receptor and progesterone receptor in the COH induced rats. In addition, after administration of BTG, the litter rate and endometrial receptivity markers, such as leukemia inhibitory factor and vascular endothelial growth factor A were increased [17]. However, the mechanisms of BTG have not been fully elucidated. The application model of network pharmacology is consistent with the overall concept of TCM. It uses integrated bioinformatics analysis to predict key molecules and pathways, and validation of the outcomes by in vivo or in vitro experiments is necessary [19].

Here, the ingredients were predicted using a combination of network database and UPLC-MS. Molecular docking technology was used for preliminary screening. Ultimately, in order to further confirm the key targets of BTG, in vivo experiments were carried out. The purpose of this study was to seek new mechanism by which BTG reserves the malignant effects of COH on endometrial morphology.

## Materials and methods

### Preparation of BTG

BTG is composed of 12 herbs: *Ligusticum chuanxiong* Hort., *Salvia miltiorrhiza* Bge., *Angelica sinensis* (Oliv.) Diels, *Eclipta prostrata* (L.) L., *Ligustrum lucidum* Ait., *Taxillus chinensis* (DC.) Danser, *Dioscorea opposita* Thunb., *Cornus officinalis* Sieb. et Zucc., *Rehmannia glutinosa* (Gaertn.) DC., *Cuscuta chinensis* Lam., *Dipsacus asper* Wall. Ex Henry, and *Hominis Placenta*. All herbs were purchased from the Guangdong Yi Fang Pharmaceutical Co., Ltd. Detailed information of the medicinal parts, meridians, and composition ratio was summarized in Table S1.

### Compounds identification and screening

UPLC-MS analysis was carried out firstly. The sample of BTG was redissolved with pure water (vortex for 30 s and ultrasonic for 3 min). L-2-chlorophenylalanine (0.06 mg/mL) as an internal standard was added and left at -20 ℃ for 2 h, and then centrifuged at 13000 rpm for 10 min at 4 ℃. Subsequently, the supernatant was passed through a 0.22 μm organic phase filer membrane, and then transferred to the injection bottle and stored at -80℃. ACQUITY UPLC I-Class system (Waters Corporation, Milford, USA) coupled with Q-Exactive quadrupole-Orbitrap mass spectrometer equipped with heated electrospray ionization (ESI) source (Thermo Fisher Scientific, Waltham, MA, USA) was applied to analyze the metabolic profiles. An ACQUITY UPLC HSS T3 column (100 mm × 2.1 mm, 1.8 μm) was used in both ESI positive and negative modes. The mobile phase A and B respectively consisted of water (containing 0.1% formic acid) and acetonitrile (containing 0.1% formic acid). The linear gradient was as followed: 0 min, 95% A; 2 min, 95% A; 4 min, 70% A; 8 min, 50% A; 10 min, 20% A; 14 min, 0% A; 15 min, 0% A; 15.1 min, 95% A, 16 min, 95% A. The injection volume was 2 μL, and the temperature was kept at 4℃. The mass scan range was from 100–1200 m/z, The resolution for HCD MS/MS scans was set at 17500. The mass spectrometer was set as follows: spray voltage, + 3.8 kV- and -3.2 kV; sheath gas flow rate, 40; aux gas flow rate, 10. The temperature was kept at 320℃.

Subsequently, the traditional Chinese medicine systems pharmacology (TCMSP) (https://old.tcmsp-e.com/tcmsp.php) was searched to screen the results of UPLC-MS. Through the evaluation of absorption, distribution, metabolism, excretion, and toxicity (ADMET), oral bioavailability (OB) and drug-likeness (DL) were identified as the indicators used to assess the main components of the compound. We set OB ≥ 30% and DL ≥ 0.18 as the screening criteria for active ingredients of drugs [20].

### Target prediction

Protein targets prediction of BTG was performed using the filtered active ingredients. Adopting the standard chemical structure obtained from the PubChem platform (https://pubchem.ncbi.nlm.nih.gov/), ingredients related targets were identified on the SwissTargetPrediction platform (http://www.swisstargetprediction.ch/). Besides, the HERB database (http://herb.ac.cn/) for direct retrieval of herbal action targets was used for prediction. Genes related to endometrial receptivity disorder after COH were screened in the Gene Expression Omnibus (GEO) database (https://www.ncbi.nlm.nih.gov/geo/). The datasets were shown in the form of volcano maps, and the intersection of the differentially expressed genes (DEG) after COH intervention and BTG prediction targets was demonstrated in the form of Venn map. The screening of DEG was carried out using the limma package of R [21], and the filtering condition were set to *p* value < 0.05 and *log*2FC >|3|. The visualization of all the above data was completed by R. Finally, a BTG-active ingredients-potential targets-disease interaction network was made by Cytoscape.

### Construction of protein–protein interaction (PPI) network

The potential targets of BTG improving endometrial receptivity after COH were used for PPI construction. We uploaded these genes to STRING (https://cn.string-db.org/), and the minimum interaction score was set to 0.4. The results were visualized by Cytoscape. In addition, the MCODE app based on vertex-weighting was applied to discover high-density regions in the graph.

### Enrichment analysis

To explore the pathways and biological function of BTG ameliorating endometrial dysfunction, the results of obtained by PPI were enriched and analyzed. Gene ontology (GO) and Kyoto encyclopedia of genes and genomes (KEGG) pathway enrichment analyses were conducted by Metascape platform (https://metascape.org/gp/index.html). The top ten items in each analysis were displayed by bubble chart.

### Molecular docking

In order to preliminarily confirm the action intensity of the screened ingredients on potential targets, the molecular docking based on the computer-assistant technology was performed. We downloaded the three-dimensional structures of molecules and proteins respectively from the PubChem platform and the PDB platform (https://www.rcsb.org/). The selection of protein tertiary structure was based on the principle that the structure is as complete as possible and the conformational resolution is low. If the results verified by experiments cannot be found, the protein conformation predicted by AlphaFold was applied [22]. After the preliminary processing of ligand and receptor, the binding energy was calculated using the AutoDock Vina software [23], and the results were shown in the form of heat map. PyMOL was used to visualize the ligand-receptor with low binding energy [24].

### In vivo experiments protocol

Eighty-two female Sprague–Dawley rats (200 ± 10 g) were purchased from Beijing Vital River Laboratory Animal Technology Co., Ltd. These animals have free access to food and water and undergo one week of adaptive breeding and estrous cycle determination. The animal experiment in this study has been approved by the Laboratory Animal Welfare Ethics branch of the Biomedical Ethics Committee of Peking University (LA2020032). All rats were randomly divided into three groups: control group (*n* = 26), model group (*n* = 28), and BTG group (*n* = 28). After the estrous cycle was confirmed by vaginal smears, the control group rats in estrus stage were mated. Pregnant mare serum gonadotrophin (PMSG) (8 IU/100 g, PROSPEC hor-272, Israel) was injected intraperitoneally to the rats in the model group and the BTG group during the diestrus stage, and HCG (16 IU/100 g, PROSPEC hor-250, Israel) was injected 48 h later. Three hours after HCG injection, rats in the two groups were mated. All female rats used for mating were caged with male rats at a ratio of 1:2 overnight, and the success of mating was confirmed by the presence of vaginal plugs the next morning. The day was designated as the first day post coitus. From our previous study, 3.27 g/kg was identified as the optimum dose of BTG in rats, and we administrated the rats in BTG group by gavage at this dose three days before diestrus stage, for ten consecutive days. All rats were sacrificed by cervical dislocation after intraperitoneal injection of 1% pentobarbital sodium at five days post coitus, one segment of the uterus was placed in 4% paraformaldehyde for morphological observation and immunohistochemistry (IHC), the other segment was stored in the -80℃ for Western blot.

### Histological evaluation with HE staining

The uterus was removed from the 4% paraformaldehyde, embedded with optimum cutting temperature compound, and cut into 8 μm sections. Subsequently, the frozen sections were dip-stained with hematoxylin solution for 4 min, washed with water, and then eluted with gradient alcohol. Followed by eosin staining solution for 1 min, dehydrated and sealed with gum. Endometrial morphology revealed by hematoxylin–eosin (HE) stained sections was assessed by the nano-Zoomer slide scanner (Japan).

### Immunohistochemistry analysis

Firstly, the frozen sections were washed with water, and the antigen was repaired with citric acid antigen repair solution. Then the endogenous peroxidase was inactivated by 3% H_2_O_2_ and the sections were rinsed with phosphate buffered saline (PBS). The sections were subsequently incubated with primary antibodies [forkhead box O1A (FOXO1A) (1:200), β-catenin (1:200), and cyclooxygenase-2 (COX-2) (1:200)] at 4 ℃ overnight, and incubated with secondary antibodies the next morning. FOXO1A (ab179450), β-catenin (ab32572), and COX-2 (ab179800) were obtained from Abcam company (UK). Based on the primary antibody source, we utilized an HRP-conjugated goat anti-rabbit secondary antibody obtained from Zhongshan Golden Bridge Biotechnology company (China). Following DAB staining, subsequent counterstaining with hematoxylin, differentiation with hydrochloric acid alcohol, and final dehydration with graded ethanol and clearing with xylene were conducted. The specimens were then mounted with neutral gum and observed using the nano-Zoomer slide scanner (Japan).

### Western blot analysis

The samples were removed from -80℃ and homogenized in RIPA buffer. The BCA assay kit was used to determine the protein concentration of the supernatant after centrifugation, and it should be ensured that the *R*-square value of the standard curve was larger than 0.99. Subsequently, the samples were subjected to a 10-well SDS-PAGE and electrotransferred to a PVDF membrane. The membranes were then blocked with 5% skim milk, followed by incubation with primary antibodies [FOXO1A (1:5000), β-catenin (1:5000), COX-2 (1:3000), and β-actin (1:10000)] at 4℃ overnight and secondary antibodies the next day. β-actin obtained from Abcam company (UK) was regarded as the internal parameter. The ECL assay kit was used to detect protein signals, and the visualization was performed by a Tanon 5200 imaging system. The expression of proteins was assessed by Image J software.

### In vitro experimental protocol

Human endometrial stromal cells (hESC) were obtained from the Meisen Chinese Tissue Culture Collections (Zhejiang, China). The basal culture medium for hESC comprised DMEM/F12 supplemented with 10% fetal bovine serum (FBS) and 1% penicillin–streptomycin. The culture environment was maintained at 5% CO2 and 37 °C. Potential compounds with the highest promise, identified from mass spectrometry and molecular docking results, were selected to assess their toxicity on hESC. Six gradients of concentrations were established to determine the IC50 of these compounds.

D-Galactose (D-Gal) was procured from Solabio Company (Beijing, China) and prepared in DMEM/F12 at a concentration of 200 mM as the modeling drug. hESC were uniformly seeded at 4 × 10^3^ cells/well in a 96-well plate with 6 replicates per group, while wells treated only with basal culture medium served as the control group. Following cell adhesion, a 24-h intervention with 200 mM D-Gal was administered, followed by a 24-h culture with the appropriate concentration of natural product selected from the screening. CCK8 solution (Solabio, Beijing, China) was added to the wells and incubated in the culture chamber for 2 h, and absorbance was recorded at 450 nm using a microplate reader (Bio-Rad, USA).

### Statistical analysis

All data in this study were analyzed by SPSS 22.0 software, and the results were presented as mean ± SEM. The one-way ANOVA, followed with Turkey’s test was performed for multiple comparison. *p* value < 0.05 was considered statistically significant. GraphPad Prism is utilized for calculating IC50 values and organizing graphs.

## Results

### Composition analysis and screening of BTG

**Fig. 1:**
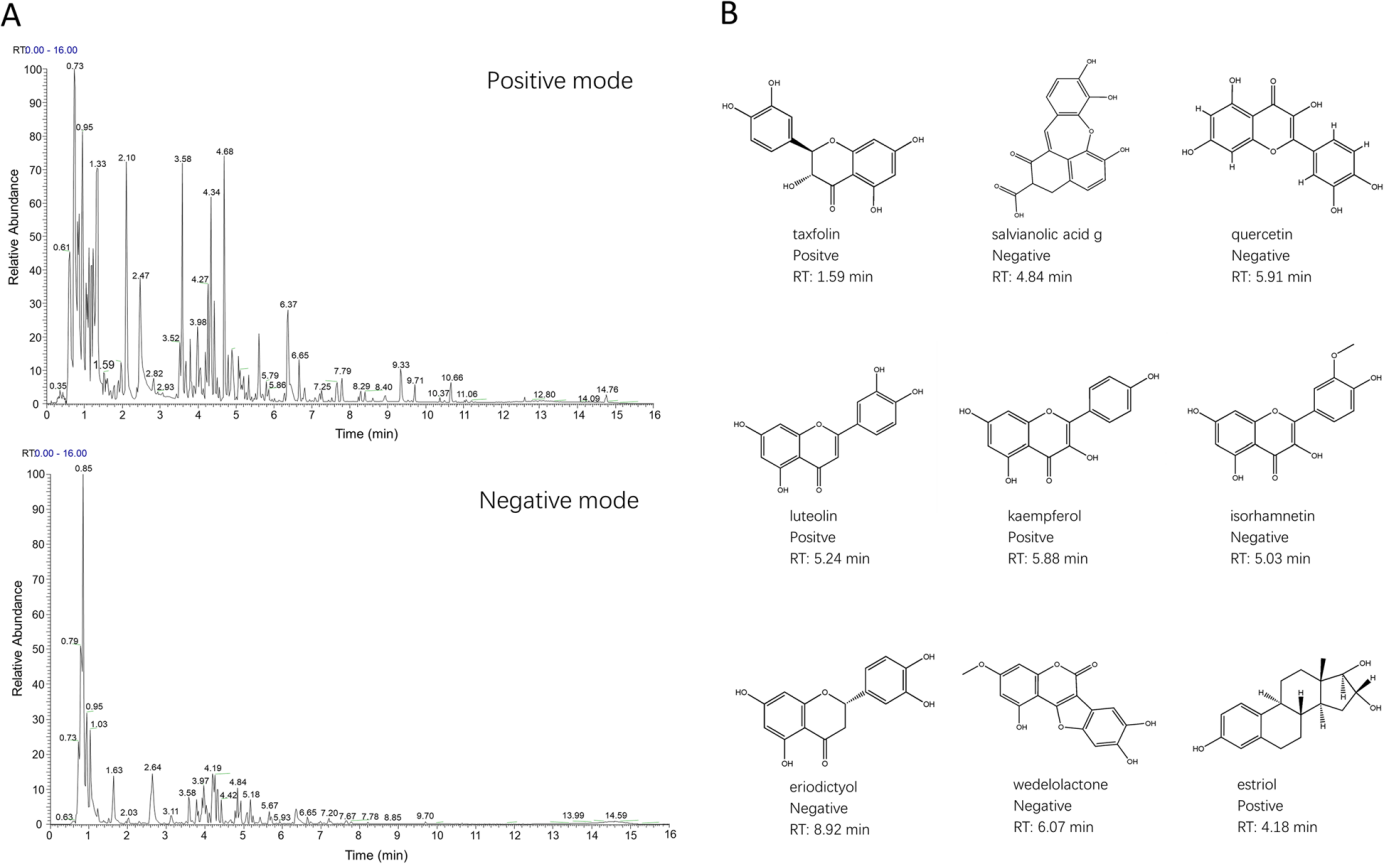
Chemical composition analysis of BTG through UPLC-MS/MS. **A** Total ion chromatogram in both positive and negative ion modes. **B** Chemical structure formula of nine screened bioactive ingredients

Through the positive and negative modes of UPLC-MS, thousands of components were detected. In order to improving the targeting and accuracy of this study, components of the 12 herbs contained in BTG were searched in the network database respectively. Using the screening conditions described previously, a total of 141 chemical constituents have been confirmed by existing literature, while only nine of them coincided with the results of UPLC-MS/MS (Fig. 1). Details of the compounds were listed in Table S2.

### Prediction of BTG targets and disease-related genes

**Fig. 2:**
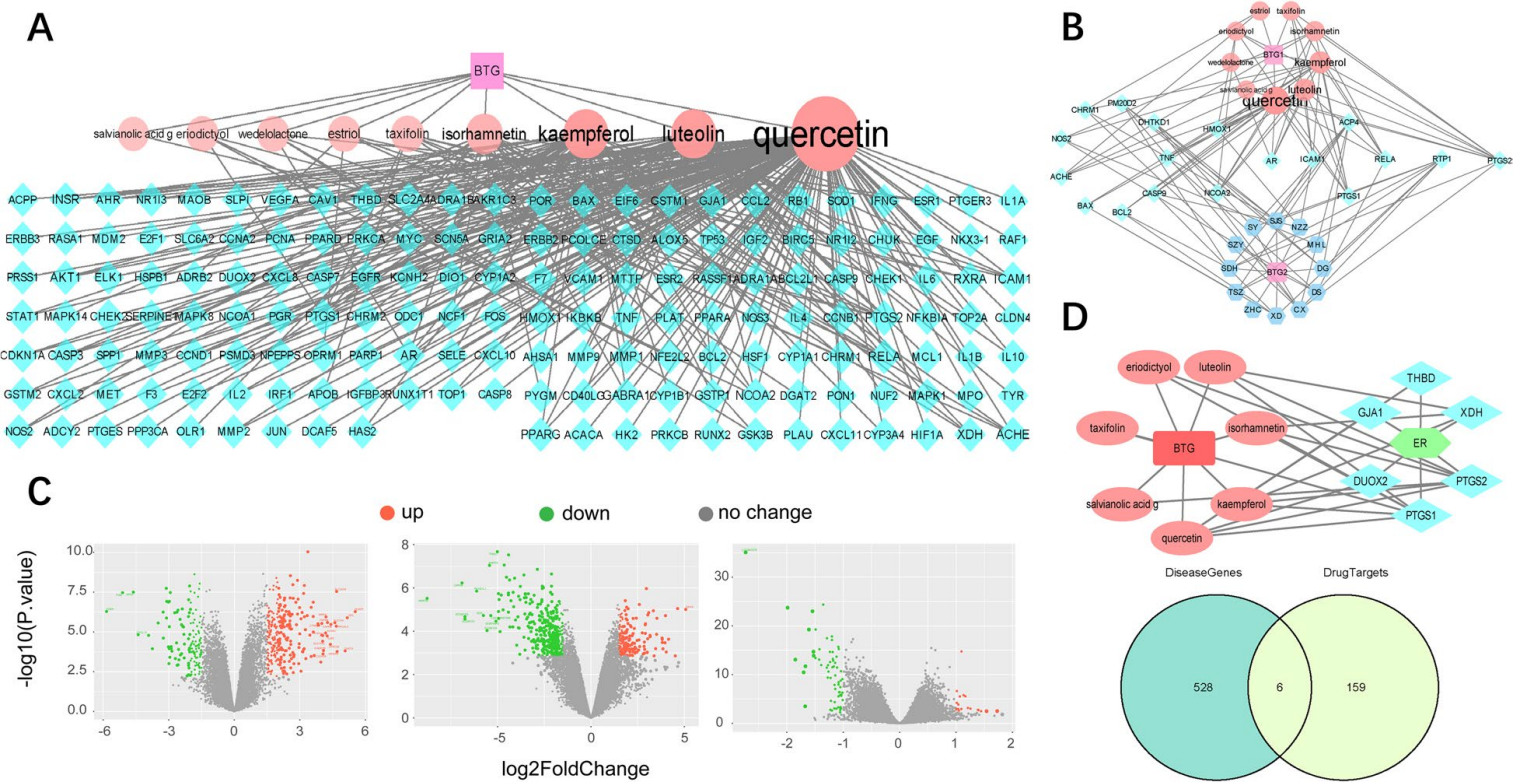
Prediction of BTG targets and COH related genes. **A** BTG-ingredients-targets network. **B** Integrated network of prediction of ingredients-targets and herbs-targets. **C** Volcano map of three datasets from GEO and Venn map of DEGs. **D** Filtered BTG-ingredients-targets network

The nine active components of BTG were input into TCMSP and SwissTargetPrediction platform to obtain the putative targets, and 165 targets were collected (Fig. 2A). In addition, HERB was used to acquire a single herbal related target. After crossing with the targets of the nine components, and the minimum value of the number of edges was set to two, eighteen targets were obtained (Fig. 2B). The GEO database was used to explore DEGs, and three datasets (GSE92324, GSE107914, and GSE165004) were considered related. Subsequently, six genes (PTGS1, PTGS2, THBD, GJA1, DUOX2, and XDH) were found by intersecting these targets with predicted potential BTG targets (Fig. 2C). However, wedelolactone and estriol were not considered to be associated with those six genes, and the network association map was performed for the seven potential components of BTG and their corresponding targets (Fig. 2D).

### PPI and enrichment analysis

**Fig. 3:**
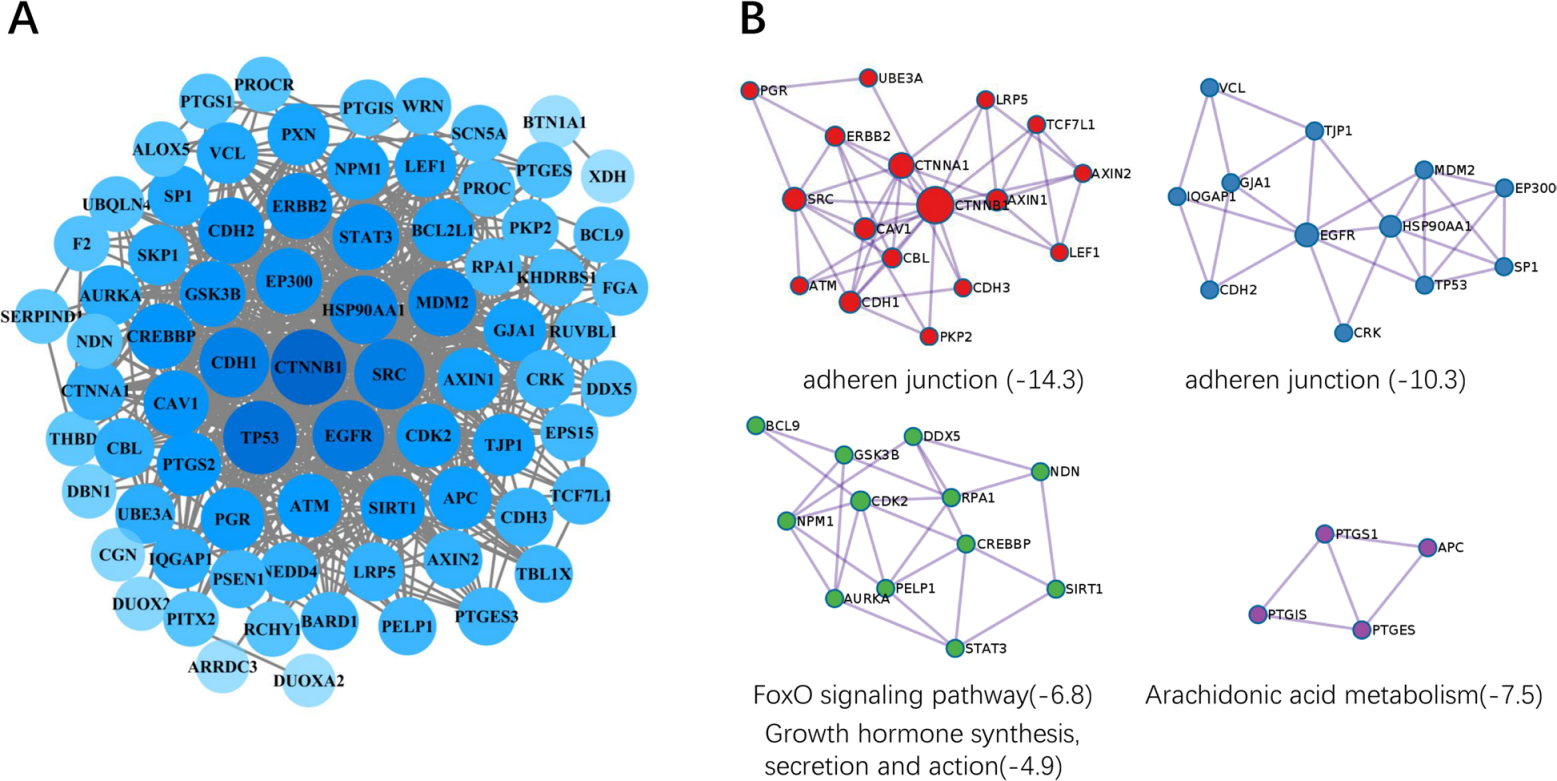
PPI construction. **A** PPI network of BTG ameliorating endometrium after COH-induced. **B** Four clustering analyses with similar functions

**Fig. 4:**
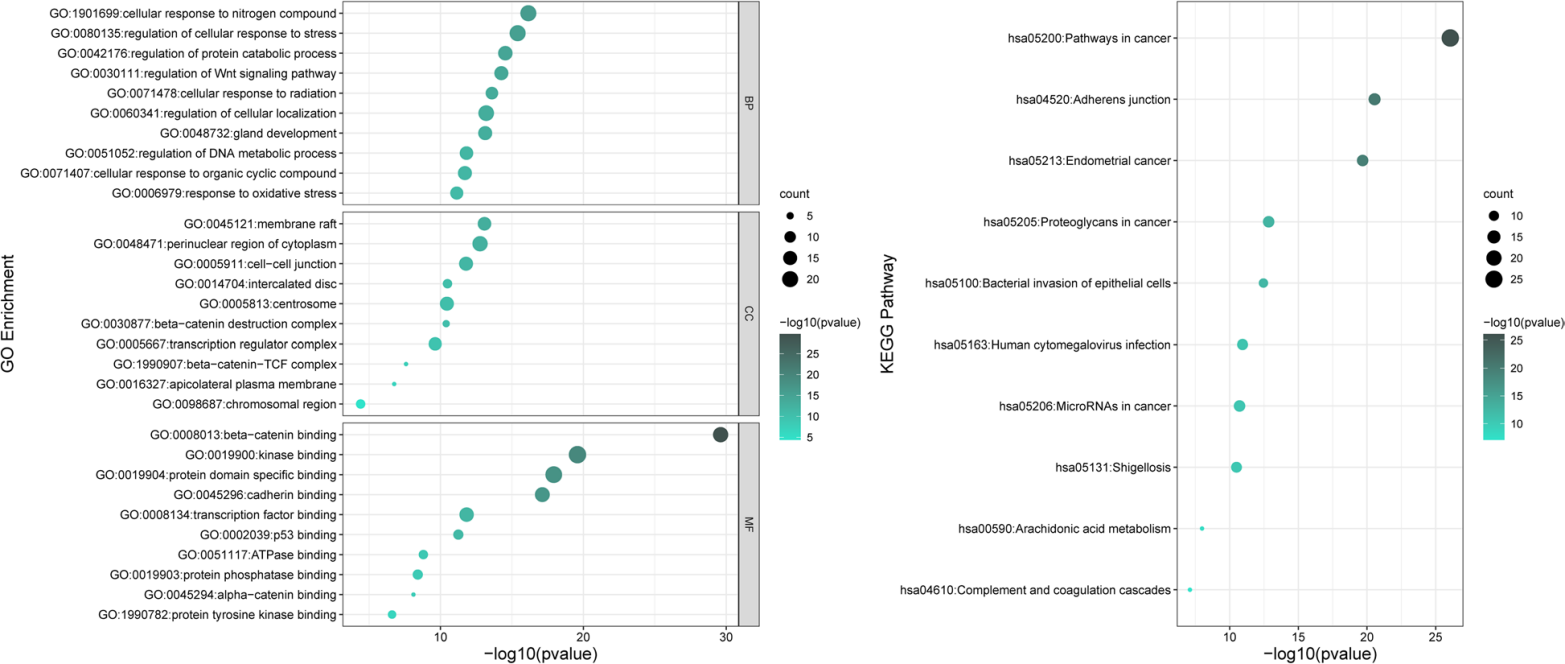
Enrichment analysis of GO and KEGG pathway

The PPI network was constructed with 76 direct or indirect potential targets, and CTNNB1, TP53, EGFR, SRC, CDH1, HSP90AA1, and MDM2 were the core genes in the PPI construction (Fig. 3A). Besides, detailed information of 15 proteins of top degrees were listed in Table 1. The MCODE analysis mainly divided the PPI into four clusters with similar functions. After excluding the heterogeneous enrichment ways such as cancer related pathways, the remaining clusters were related to adheren junction, FoxO signaling pathway, growth hormone synthesis, secretion and action, and arachidonic acid metabolism (Fig. 3B). Subsequently, GO and KEGG pathway enrichment analysis were conducted with 76 targets (Fig. 4). GO analysis consisted of biological process (BP), cellular components (CC), and molecular function (MF). The ameliorating effects of BTG on reversing COH were mainly concentrated in cellular response to nitrogen compound, regulation of cellular response to stress, and protein catabolic process. KEGG pathway enrichment analysis revealed that adheren junction, arachidonic acid metabolism, and complement and coagulation cascades, etc. were regulated by BTG.

**Table 1:** Top 15 targets in the PPI

Gene symbol	Degree	Betweenness centrality
CTNNB1	50	0.12349836
TP53	45	0.09505992
EGFR	40	0.03992262
SRC	38	0.07418278
CDH1	37	0.05107833
HSP90AA1	34	0.04285484
MDM2	33	0.03552089
CDH2	29	0.02529622
EP300	29	0.02025499
STAT3	29	0.01413901
ERBB2	29	0.01584698
GSK3B	28	0.01949055
CREBBP	27	0.01522443
CAV1	27	0.03543134
PTGS2	25	0.08776566

### Molecular docking validation

**Fig. 5:**
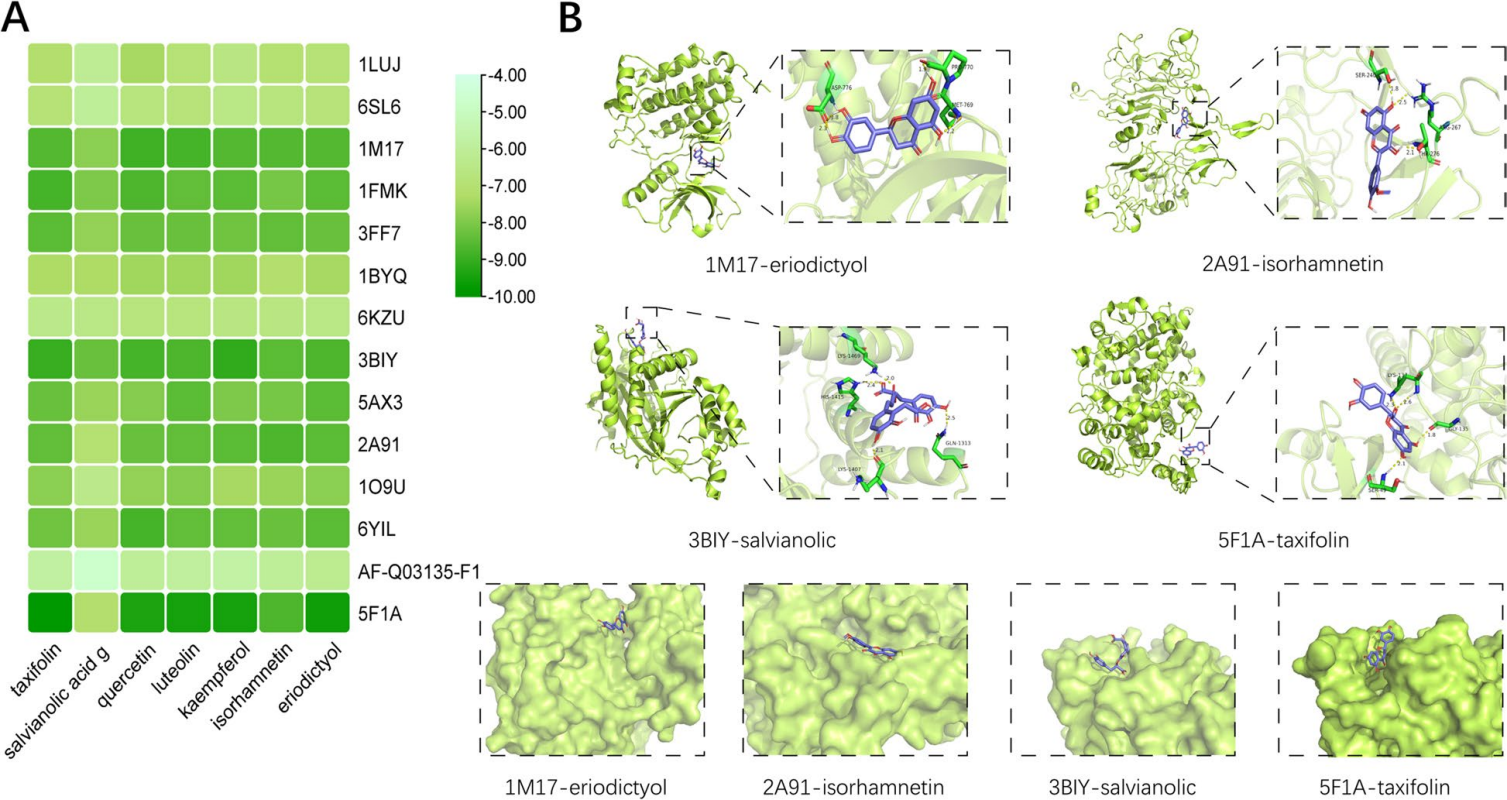
Visualization of molecular docking results. **A** Heat map of affinity of 68 molecular docking. **B** Potential binding sites and pockets of four groups of ligand-receptor docking

The targets with high degrees in the PPI network were screened out for molecular simulation docking analysis with seven potential targets. Crystal structure of targets were retrieved from the PDB database, CTNNB1 (1LUJ), TP53 (6SL6), EGFR (1M17), SRC (1FMK), CDH1 (3FF7), HSP90AA1 (1BYQ), MDM2 (6KZU), EP300 (3BIY), STAT3 (5AX3), ERBB2 (2A91), GSK3B (1O9U), CREBBP (6YIL), and PTGS2 (5F1A) were determined by X-ray diffraction, nuclear magnetic resonance, or 3D electron microscope reconstruction. The conformation of CAV1 was not determined experimentally, the structure predicted by AlphaFold was applied (AF-Q03135). The affinity was considered as the sign of docking strength, and detailed docking results were shown in Table S3. Each of the affinity was less than -5.8 kcal/mol, and the average was -7.75 kcal/mol (Fig. 5A). Besides, four well-combined patterns were selected for visualization in Fig. 5B.

### BTG ameliorates endometrial morphology of COH rats during implantation

**Fig. 6:**
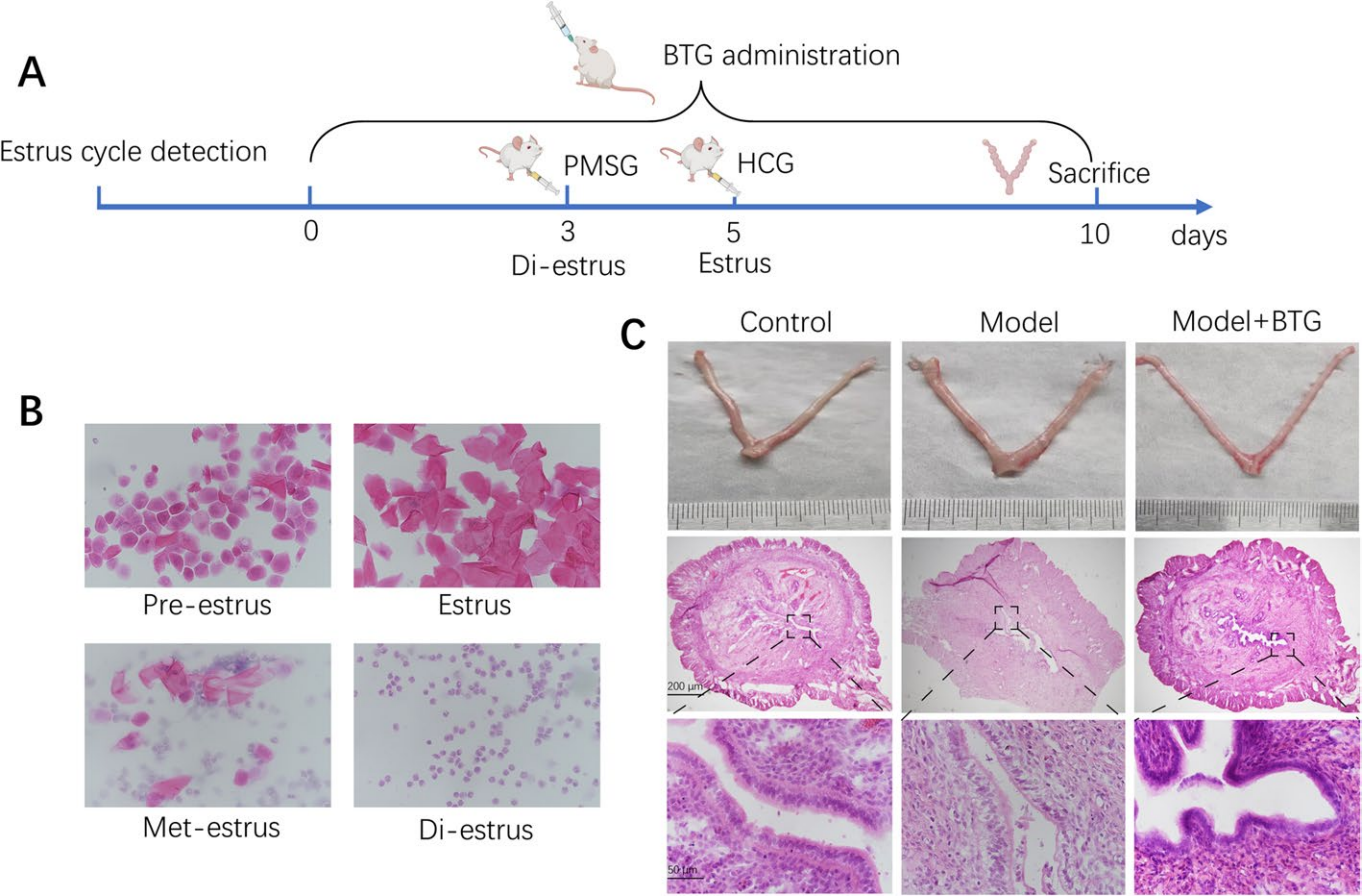
Experiment protocol and display of pathology. **A** Experimental flow chart. **B** Typical characteristics of estrus cycle. **C** Endometrial appearance and sections of different times of three groups of rats (5 × and 40 ×)

The experimental protocol was shown in Fig. 6A. Before modeling, the estrus cycle was first confirmed. Figure 6B showed the typical characteristics of the four cycles. Compared with the control group, the fresh uterus obtained from the sacrificed COH rats was characterized by obvious edema (Fig. 6C). HE staining was then performed to evaluate the endometrial morphology. Figure 6C showed that the endometrial epithelial cells were arranged orderly, and the fibroblasts in the stroma were decidualized in the rats of control group. While in the COH rats, the uterus epithelial cells were loosely arranged, the cell edema was obvious, and the decidua of stroma cells was not significant, which affected the embryo implantation. Administration of BTG reversed the loose and edema structure of epithelial cells, enhanced the secretory activity, enriched blood vessels, and finally ameliorated endometrial dysfunction.

### BTG reverses the decreased protein expression of FOXO1A, β-catenin, and COX-2

**Fig. 7:**
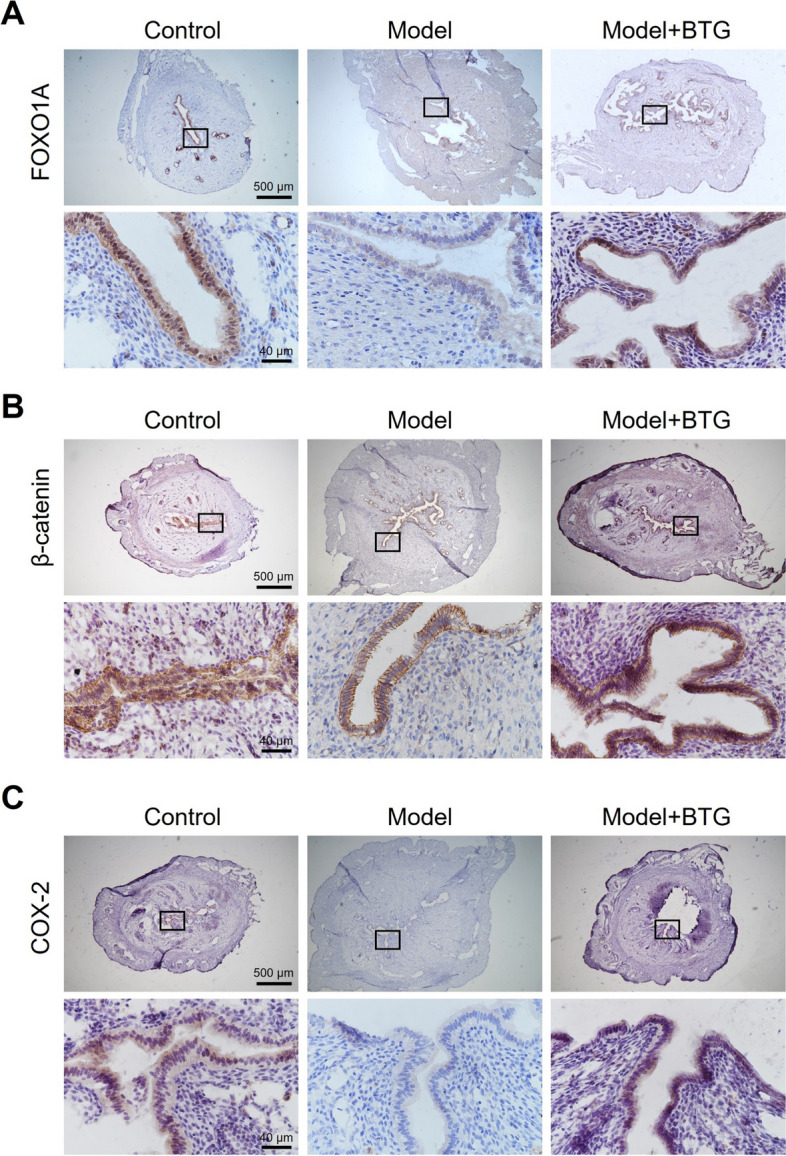
IHC results (5 × and 40 ×) of BTG on the protein expression of **A** FOXO1A, **B** β-catenin, and **C** COX-2 in COH-induced rats

**Fig. 8:**
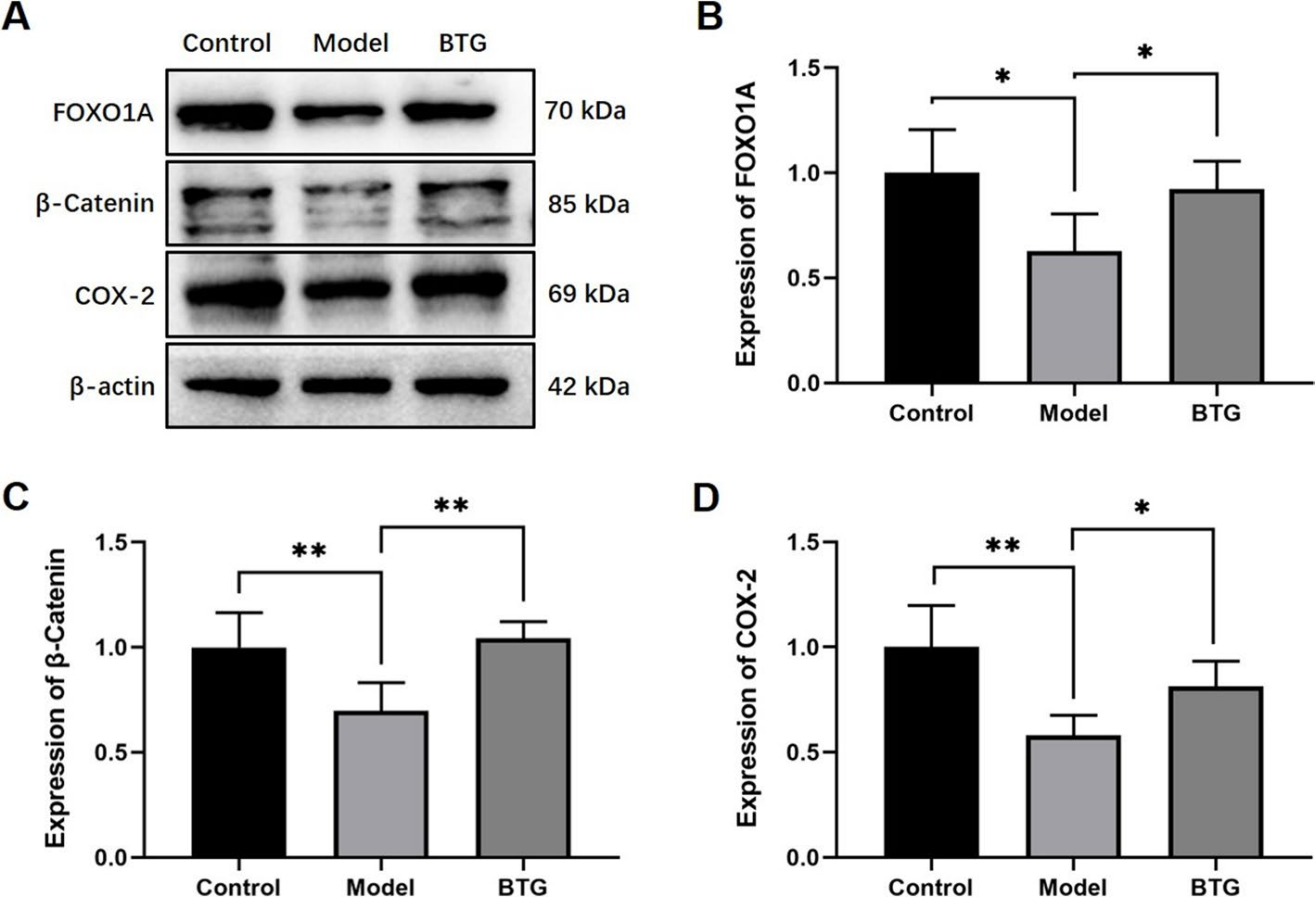
Western blot results of BTG on the protein expression of FOXO1A, β-catenin, and COX-2 in COH-induced rats (**A**-**D**). ^*^p < 0.05 and.^**^p < 0.01

Differences in expression of protein levels were verified by IHC and western blot. Figure 7A showed that FOXO1A was expressed in the luminal epithelium and glandular epithelium of the endometrium in both the three groups of rats. After COH, the expression of FOXO1A decreased, but increased with BTG administration. Beta-catenin was distributed in the cell membrane and cytoplasm, and its apical expression was obvious after COH. BTG administration increased its expression (Fig. 7B). In the endometrium of the three groups, expression of COX-2 was little, but BTG administration increased the trend of decrease in COH rats (Fig. 7C). In order to compensate for the insufficient feasibility in quantification of IHC, western blot was conducted to quantify expression of these three proteins. Compared with the control group, the expression of FOXO1A ($p<0.05$), β-catenin ($p<0.01$), and COX-2 ($p<0.01$) was decreased in the model group. BTG administration increased the expression of FOXO1A ($p<0.05$), β-catenin ($p<0.01$), and COX-2 ($p<0.05$) in the rats after COH (Fig. 8).

### The cytotoxicity of D-Gal and potential active components in BTG on HESCs

**Fig. 9:**
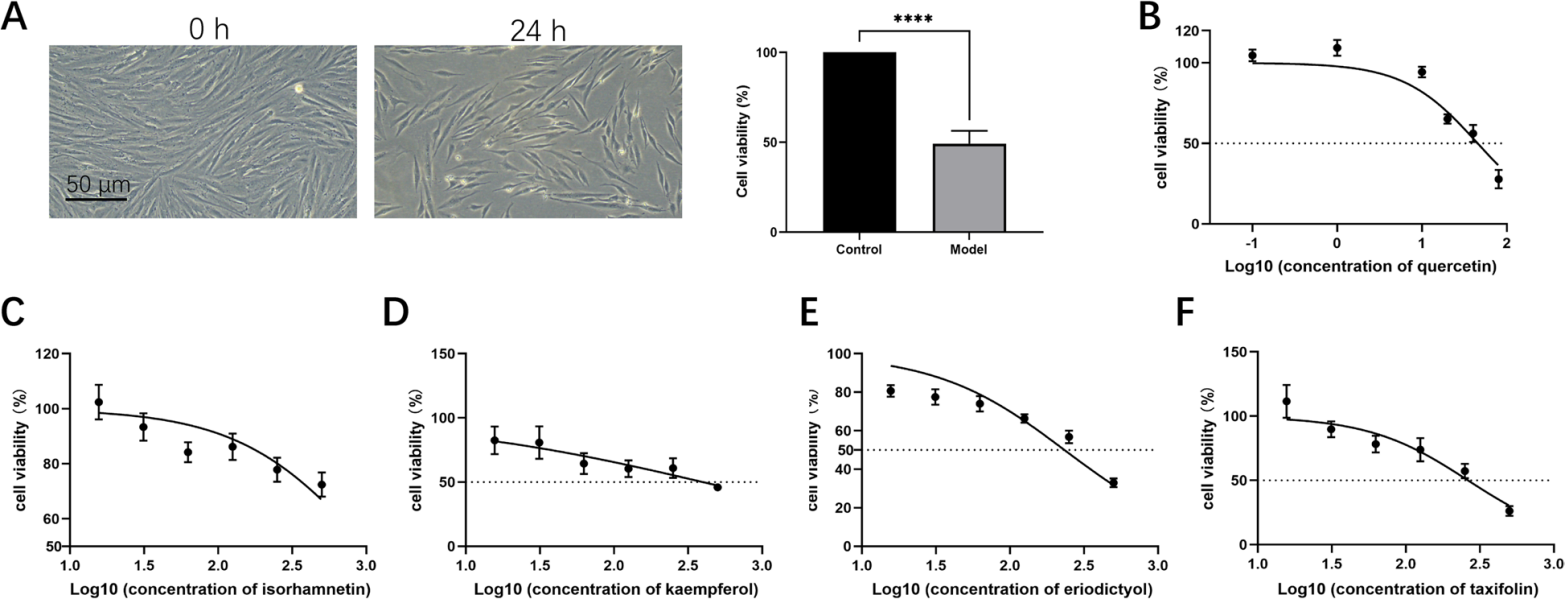
Cytotoxicity of D-Gal and 5 natural products on hESCs. **A** D-Gal inhibited the cell viability of hESCs (10 ×). The effect of concentration gradients of **B** quercetin, **C** isorhamnetin, **D** kaempferol, **E** eriodictyol, and **F** taxifolin on the cell viability of hESCs. ****p < 0.0001

Previous studies have identified an increased senescent phenotype in endometrial stromal cells post COH, which is unfavorable for decidualization during embryo implantation. D-Gal is a widely recognized mimetic agent causing oxidative stress damage and promoting aging. Results demonstrate that 200 mM of D-Gal induced nearly 50% inhibition of cell proliferation in hESCs (Fig. 9A). Subsequently, quercetin, taxifolin, kaempferol, eriodictyol, and isorhamnetin were selected based on mass spectrometry and molecular docking results for further exploration of their protective effects on fertility. Concentration gradients of 6 levels were set for each compounds according to existing literature: quercetin at concentrations of 0.1 μM, 1 μM, 10 μM, 20 μM, 40 μM, and 80 μM; taxifolin, kaempferol, eriodictyol, and isorhamnetin at concentrations of 15.625 μM, 31.25 μM, 62.5 μM, 125 μM, 250 μM, and 500 μM, respectively. As illustrated in Fig. 9B-F, even at a concentration of 500 μM, isorhamnetin failed to achieve 50% inhibition of hESC proliferation. The IC50 values were determined as follows: quercetin, 45.35 μM; taxifolin, 263.1 μM; kaempferol, 398.9 μM; eriodictyol, 233.2 μM. These findings suggest that these potential natural products exhibit limited cytotoxicity towards hESCs.

### Protective effects of 5 potential compounds against D-Gal-induced damage in hESC

**Fig. 10:**
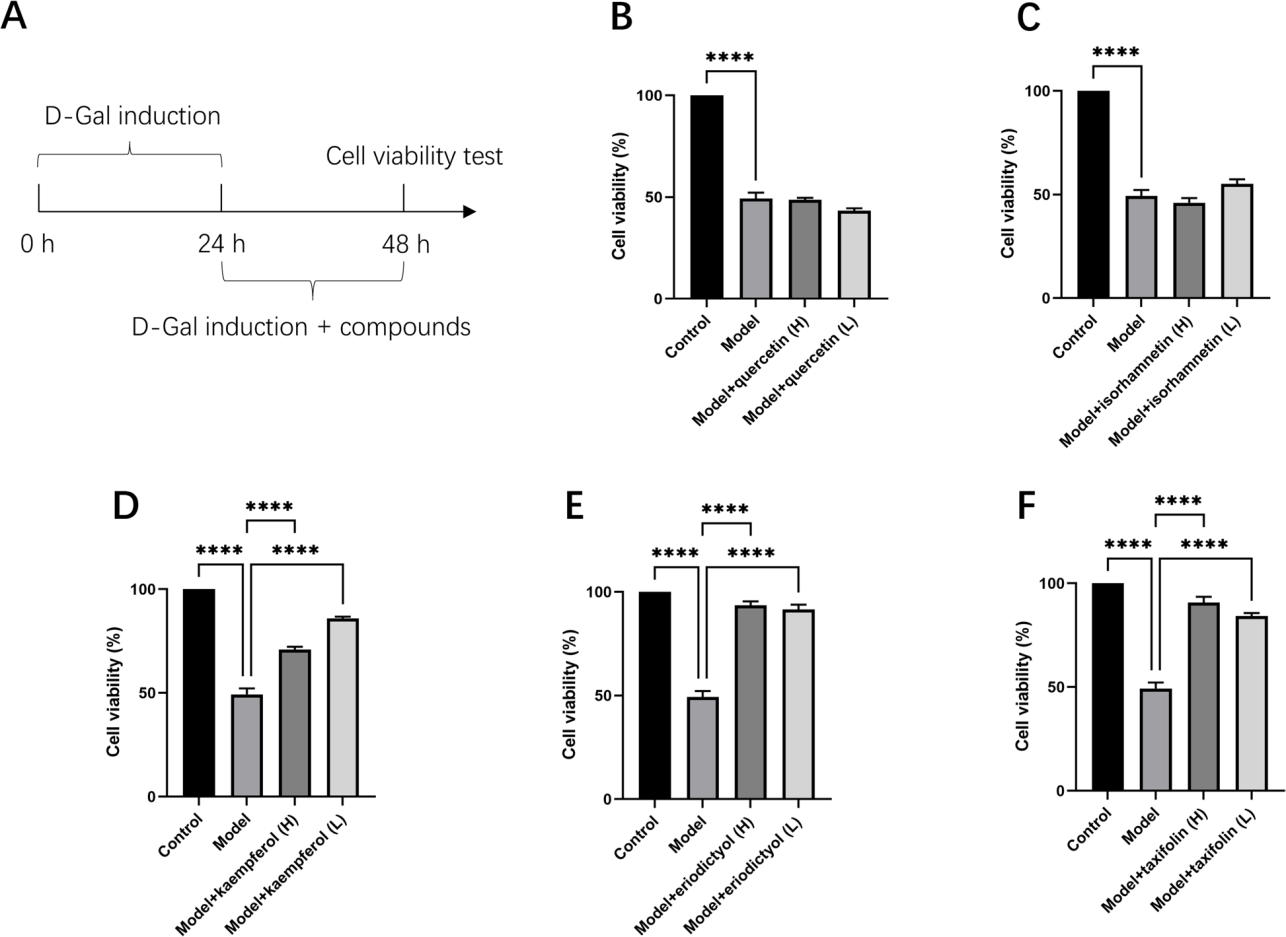
Protective effects of 5 compounds from BTG against D-Gal-induced impairment of hESC cell viability. **A** Flowchart depicting the modeling of D-Gal and compounds interventions. **B** Quercetin, **C** isorhamnetin, **D** kaempferol, **E** eriodictyol, and **F** taxifolin exhibit their effects on improving cell viability post-D-Gal modeling. ^****^p < 0.0001

Following a 24-h D-Gal intervention, two different concentrations of quercetin (50 μM and 25 μM), taxifolin (260 μM and 130 μM), kaempferol (400 μM and 200 μM), eriodictyol (230 μM and 115 μM), and isorhamnetin (500 μM and 250 μM) were separately administered for 24 h. Cell viability was assessed using CCK-8 assay (Fig. 10A). The results indicated that both quercetin and isorhamnetin failed to effectively ameliorate the detrimental effects of D-Gal. However, both concentrations of kaempferol, eriodictyol, and taxifolin exhibited an effective improvement in the cellular viability damage caused by D-Gal in hESC (Fig. 10B-F).

## Discussion

Patients with COH have various basic diseases, and the structural and functional characteristics of their endometrial environment are also heterogeneous, which requires attention to the goal of adapting measures to individual conditions in their treatment strategies [25]. Endometrial receptivity is mainly driven by progesterone and estrogen exposure, and when 2–10 days cell embryos are transplanted, progesterone exposure less than 1 day or more than 5 days will lead to implantation failure [26]. Hormone replacement therapy-frozen embryo transfer (HRT-FET) was proposed to ensure the consistency of embryo and endometrium preparation. A current prospective cohort study demonstrated that according to periodic hormone detection, individualized subcutaneous progesterone injection to patients with low progesterone the day prior to euploid FET can be unaffected by COH and ensure better ongoing pregnancy rate and live birth rate [27]. However, two meta-analyses have found that although the risk of ovarian hyperstimulation syndrome is lower for FET, the risk of pregnancy-induced hypertension and pre-eclampsia for FET is higher [28, 29]. The therapeutic strategy of complementary and alternative medicine is worth exploring.

BTG is a formula granule of TCM, and its efficacy of improving pregnancy rate on infertile patients has been proven in clinics. Our previous study has found that BTG can increase the live birth rate of COH rats, alleviate angiogenesis, and promote the decidualization of endometrial stroma in COH rats [17]. Additionally, we confirmed minimal hepatic and renal toxicity as well as a high safety profile associated with BTG administration (Fig. S2). In order to deeply explore the mechanism of BTG ameliorating endometrial receptivity, discover potential pharmaceutical targets and molecules that might be used as drugs, a network pharmacology study base on UPLC-MS, molecular docking verification and experimental validation was performed. In the past few years, network pharmacology has been widely allied in the study of TCM [30]. Its realization of constructing the interaction network between bioactive compounds and targets and the interaction between targets provides an important method for the research of TCM [31, 32]. However, some recent studies have only screened a single or a few network databases, and the results are repetitive and ineffective. Only by performing the integration of other experiments such as mass spectrometry, molecular docking, and molecular biology validation can the results of network pharmacology be more reliable [33].

The results of UPLC-MS in this study showed that thousands of metabolic components of BTG have been screened out, but after comparison with the existing literature, it was found that only nine components overlapped, which was beyond our expectation. One of the possible reasons is that the database is not updated in time. Among the nine ingredients, quercetin and kaempferol, as “panacea”, were listed as the main active ingredients by many other network pharmacology studies targeting different Chinese medicines which may be explained by the high oral bioavailability (46.43% and 41.88%) and the large number of studies [34]. Six of these nine compounds belong to flavonoids, As plant secondary metabolites widely distributed in various fruits and vegetables, flavonoids are characterized by antioxidation and have the ability to scavenge free radicals and inhibit metal ion chelators [35]. It has been demonstrated that baicalin, as a monomer of flavonoids, can increase the adhesion rate and implantation rate of mouse embryos by activating Wnt/β-catenin signaling pathway and elevating fucosyltransferase IV [36]. Besides, more and more evidence revealed that flavonoids can inhibit inflammation related regulatory enzymes and transcription factors, and the efficacy in reproductive endocrine diseases such as menopausal syndrome, endometriosis and polycystic ovary syndrome are gradually being elucidated [37, 38]. As a kind of coumarins, wedelolactone can ameliorate the Keap1/Nrf2/ARE signaling pathway, which is the central defense system of antioxidant stress, and play an active anti-inflammatory role [39]. Salvianolic acids are common in the study of vascular dysfunction and vascular inflammation [40]. They exert antioxidant stress, antithrombotic and other functions against a variety of vascular cell types [41]. As a potential component of BTG in regulating endometrial receptivity, it may improve embryo implantation by ameliorating uterine microcirculation [42]. Estriol, as a natural plant hormone, has little effect on the uterine entity and endometrium. It has feedback inhibitory effect on the hypothalamus and pituitary [43, 44]. However, up to date, no study has found whether estriol can inhibit the hormone accumulation after COH. Because wedelolactone and estriol did not find any association between their potential targets and the disease-related genes, they were excluded from the ingredient-target network. Their potential beneficial effects on endometrial receptivity cannot be denied, and should be further verified by experiments.

From the three datasets of GEO database, 524 DEGs were detected, six of which were intersected with the drug targets. After PPI construction, four clusters with similar function were shown, including adheren junction, FoxO signaling pathway, growth hormone synthesis, secretion and action, and arachidonic and metabolism. The results of GO and KEGG analysis demonstrated that BTG's regulatory effect mainly occurs in membrane rafts, participates in reactions to nitrogen compounds, cell stress reactions and protein catabolic processes, and regulates Wnt pathway. Specifically, it may be related to β-catenin binding, and enzyme binding, etc.

**Fig. 11:**
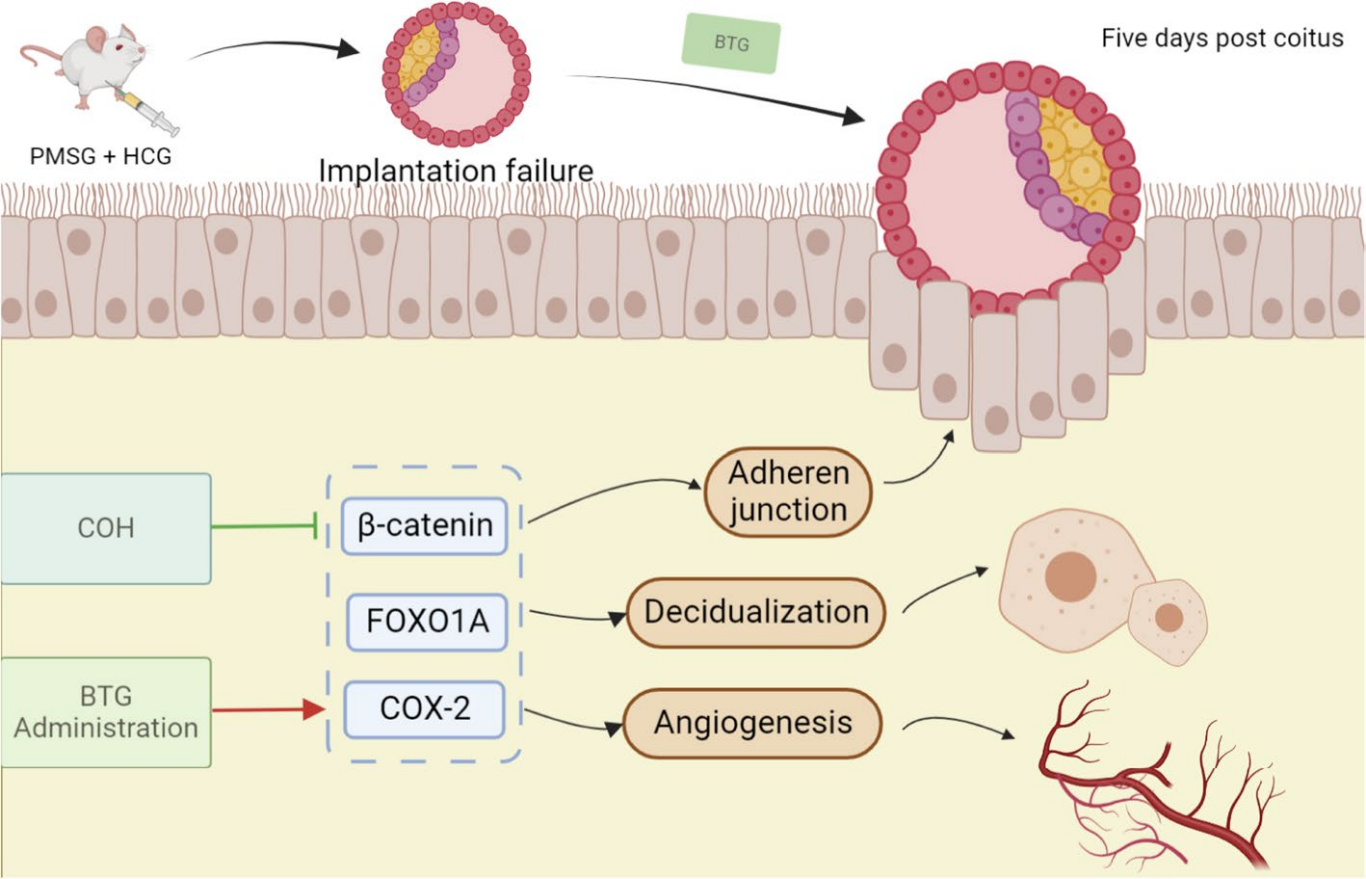
Potential mechanisms of BTG ameliorating the implantation status of endometrium after COH-induced. The green line represents inhibition and the red line represents promotion

Beta-catenin is a multifunctional catenin, which plays a role as a signal transduction factor of Wnt pathway between cells and participates in many processes such as cell adhesion, proliferation, embryonic development and tissue homeostasis [45]. In the secretory period, progesterone inhibits the effect of estrogen, making the expression of β-catenin decreased and promoting the change of endometrial status, which is also the reason for the decrease of its level after COH [46]. It was found that the expression of chromodomain Y like (CDYL) decreased in the endometrial tissues of recurrent implantation failure women, and in CDYL-knockdown human endometrial lshikawa cells, the β-catenin expression was reduced which resulted in the inhibition of cell migration ability [47]. During embryo implantation, β-catenin forms an anchor with E-cadherin on the skeleton and membrane protein, and through the combination of β-catenin and actin the stabilization of cellular connexin is promoted, which is convenient for embryos to find the implantation site [48]. While at the late stage of implantation, the expression of β-catenin is decreased, which is likely to avoid excessive invasion of embryonic trophoblast cells into the endometrium [49]. Our results showed that the expression of β-catenin in endometrium of rats decreased after COH, and the endometrial state may have missed implantation, while BTG prolongs or regulates this process. FOXO1A is a decidual marker in endometrial stromal cells, which regulates the transcription of decidual prolactin and insulin-like growth factor binding protein 1. It is dynamically expressed in mouse uterine tissue, and the expression of FOXO1A increases at implantation, which is regulated by both estrogen and progesterone [50]. At five days post coitus, FOXO1A in luminal epithelial cells prevents apoptosis in the embryo sac attachment area during endocytosis. When the uterus is in the delayed implantation state, FOXO1A is only expressed in the endometrial vessels [51]. A uterine transcriptomics study found that after FOXO1A ablation, the polarity of luminal epithelial cells changed, and embryos could not pass through the luminal epithelium, resulting in infertility. At the meanwhile, genes related to cell invasion, molecular transport, apoptosis and CTNNB1 signaling pathway were altered [52]. FOXO1A is also like a navigation molecule for embryonic localization, but its specific mechanism has not been clarified. Our results showed that FOXO1A expression was reduced after COH, but increased after BTG administration. Another verified molecule was COX-2, which is a core enzyme mediating the synthesis of prostaglandins from arachidonic acid. COX-2 can be induced by cytokines and growth factors, and its main function is to regulate inflammation and angiogenesis. A study used nimesulide as a selective COX-2 inhibitor to model mice, when the dose of nimesulide was higher than 800 μg, the implantation sites were then reduced [53]. It was also found that after COH, COX2-PGE2 pathway was inhibited and angiogenesis was reduced, which might be related to the decrease of HIF-α [54]. The results of our study demonstrated that BTG could increase the expression of COX-2 after COH. The potential mechanisms by which BTG ameliorates endometrial status after COH were summarized in Fig. 11.

To delve deeper into the pharmacokinetics of BTG, we initially conducted in vitro experiments. Five potential compounds were identified based on mass spectrometry and molecular docking results. These compounds were evaluated on hESC subjected to D-Gal modeling to validate their capacity to ameliorate cellular activity impairment. Results demonstrated significant efficacy of kaempferol in improving cell viability. Subsequent examination of kaempferol concentration gradients in the blood following BTG administration revealed sustained detectable concentrations even at 8 h, suggesting kaempferol as a pharmacologically active component underlying BTG’s effects (Fig. S2). Further exploration of additional potential fertility-enhancing compounds is warranted.

This study focused on exploring more mechanisms of BTG ameliorating endometrial morphology in rats after COH. The main limitation is that drug target prediction and DEGs were all obtained from online databases, which may exist some heterogeneities. In order to achieve more in-depth and innovative findings, multi-omics studies of TCM should be the next step.

## Conclusion

In this study, the potential bioactive ingredients of BTG were identified by UPLC-MS combined with online databases, and the mechanisms of BTG ameliorating endometrial morphology after COH was explored using systematic pharmacological methods. It was confirmed by experimental validation that BTG reversed the depressed expression of FOXO1A, β-catenin, and COX-2 after COH, reduced the edema of endometrial epithelial cells, and attenuated abnormal morphology of endometrium during the window of implantation.

### Supplementary Information

**Additional file 1: Table S1.** Composition of the BTG. **Table S2.** Identification of chemical components in BTG. **Table S3.** Affinity of ingredients with potential targets. **Figure S1.** The blood drug concentration of kaempferol following BTG administration. Kaempferol standard was obtained from MedChemExpress (USA) with a purity of 99.86% (HY-14590). An appropriate amount of kaempferol was added to 75% methanol to prepare a stock solution of 200 μg/mL for the mixed control solution. Upon usage, it was sequentially diluted to obtain concentration gradient solutions. Utilizing the same 50 female SD rats as employed in the in vivo experiment, two rats constituted the blank control group, while the remaining 48 rats were divided into treatment groups, each consisting of 6 rats. The treatment groups received oral gavage of BTG at 3.27 g/kg for 3 consecutive days, while the blank control group received an equivalent volume of physiological saline using the same procedure. Prior to the final dosing, the rats were fasted for 12 hours but allowed access to water. Blood samples were collected from the abdominal aorta of the rats at 8 time intervals post administration (10 min, 20 min, 30 min, 1 h, 2 h, 4 h, 8 h, 12 h) after anesthetizing the rats using 1% pentobarbital sodium. The blood samples were centrifuged at 4°C, 3500 rpm for 10 min, and the supernatant was analyzed. Following the UPLC-MS analysis method described earlier, the peak areas of the analyte and internal standard were recorded to calculate the blood drug concentrations at each time point. **Figure S2.** The impact of BTG administration on rat liver and kidney function. The serum levels of (A) ALT, (B) AST, (C) CREA, (D) UA, and (E) UREA in each group of rats were measured using microplate assays. The assay kits were obtained from Nanjing Jiancheng Bioengineering Institute (China).

## Data Availability

Additional data are available upon request from the corresponding authors.
