# Supplementary material for: Mechanisms of Bushen Tiaoxue Granules against controlled ovarian hyperstimulation-induced abnormal morphology of endometrium based on network pharmacology
Source: J Ovarian Res. 2024 Jan 26;17:25. doi: 10.1186/s13048-023-01339-3 (PMC10811918; doi:10.1186/s13048-023-01339-3)

SUPPLEMENTARY DATA

Table S1. Composition of the BTG.

Herb pinyin name	Herb Latin name	Use part	Meridians	Percentage of composition ratio
Chuanxiong	Radix chuanxiong	rhizome	Liver; Cardiovascular; Gallbladder	7.7
Danshen	Radix Salviae liguliobae	root	Liver; Heart	9.6
Danggui	Radix Angelicae sinensis	root	Spleen; Liver; Heart	10.7
Mohanlian	herba ecliptae	aerial parts	Liver; Kidney	2.9
Nvzhenzi	Fructus Ligustri lucidi	fruit	Liver; Kidney	2.5
Sangjisheng	Herba Taxilli	stem and branch-leaf	Liver; Kidney	4.3
Shanyao	Rhizoma Dioscoreae	rhizome	Lung; Spleen; Kidney	5.5
Shanzhuyu	Fructus Corni	fruit	Liver; Kidney	16.1
Shudihuang	Radix Rehmanniae Preparata	steamed and sundried root	Liver; Kidney	13.4
Tusizi	Semen Cuseutae;Semen Cuseutae	seed	Liver; Kidney	3.7
Xuduan	Radix Dipsaci	root	Liver; Kidney	5.9
Ziheche	Homo sapiens	human placenta	Lung; Liver; Heart; Kidney	17.8

Table S2. Identification of chemical components in BTG.

Retention Time/min	Formula	Metabolites	Molecular Weight/Da	Ion Mode	Mass Error/ppm
1.59	$C_{15}H_{12}O_7$	taxifolin	304.25	pos	1.25
4.84	$C_{18}H_{12}O_7$	salvianolic acid g	340.3	neg	1.24
5.91	$C_{15}H_{10}O_7$	quercetin	302.23	neg	1.01
5.24	$C_{15}H_{10}O_6$	luteolin	286.23	pos	1.23
5.88	$C_{15}H_{10}O_6$	kaempferol	286.23	pos	-1.27
5.03	$C_{16}H_{12}O_7$	isorhamnetin	316.26	neg	1.80
8.92	$C_{15}H_{12}O_6$	eriodictyol	288.25	neg	1.05
6.07	$C_{16}H_{10}O_7$	wedelolactone	314.24	neg	1.95
4.18	$C_{18}H_{24}O_3$	estriol	288.38	pos	0.17

Table S3. Affinity of ingredients with potential targets.

Ligand	Receptor	PDB ID	Affinity/(kcal/mol)
taxifolin	CTNNB1	1LUJ	-7.1
	TP53	6SL6	-6.9
	EGFR	1M17	-8.6
	SRC	1FMK	-8.8
	CDH1	3FF7	-8.5
	HSP90AA1	1BYQ	-7.3
	MDM2	6KZU	-6.4
	EP300	3BIY	-9
	STAT3	5AX3	-8.3
	ERBB2	2A91	-8.4
	GSK3B	1O9U	-7.8
	CREBBP	6YIL	-8.2
	CAV1	AF-Q03135-F1	-5.8
	PTGS2	5F1A	-9.8
salvianolic acid g	CTNNB1	1LUJ	-6.1
	TP53	6SL6	-6
	EGFR	1M17	-7.8
	SRC	1FMK	-8
	CDH1	3FF7	-7.7
	HSP90AA1	1BYQ	-7.3
	MDM2	6KZU	-6.5
	EP300	3BIY	-8.3
	STAT3	5AX3	-7.6
	ERBB2	2A91	-7
	GSK3B	1O9U	-6.4
	CREBBP	6YIL	-7.6
	CAV1	AF-Q03135-F1	-4.7
	PTGS2	5F1A	-7.1
quercetin	CTNNB1	1LUJ	-7.4
	TP53	6SL6	-6.7
	EGFR	1M17	-8.9
	SRC	1FMK	-8.7
	CDH1	3FF7	-8.3
	HSP90AA1	1BYQ	-7.5
	MDM2	6KZU	-6.7
	EP300	3BIY	-8.8
	STAT3	5AX3	-7.9
	ERBB2	2A91	-8.3
	GSK3B	1O9U	-7.7
	CREBBP	6YIL	-8.8
	CAV1	AF-Q03135-F1	-6
	PTGS2	5F1A	-9.4

luteolin	CTNNB1	1LUJ	-6.9
	TP53	6SL6	-6.8
	EGFR	1M17	-8.8
	SRC	1FMK	-8.4
	CDH1	3FF7	-8.3
	HSP90AA1	1BYQ	-7.5
	MDM2	6KZU	-6.7
	EP300	3BIY	-8.7
	STAT3	5AX3	-8.5
	ERBB2	2A91	-8.4
	GSK3B	1O9U	-7.9
	CREBBP	6YIL	-8.4
	CAV1	AF-Q03135-F1	-5.9
	PTGS2	5F1A	-9.5
kaempferol	CTNNB1	1LUJ	-6.6
	TP53	6SL6	-6.5
	EGFR	1M17	-8.6
	SRC	1FMK	-8.5
	CDH1	3FF7	-8.2
	HSP90AA1	1BYQ	-7.5
	MDM2	6KZU	-6.6
	EP300	3BIY	-9.1
	STAT3	5AX3	-8
	ERBB2	2A91	-8.5
	GSK3B	1O9U	-7.4
	CREBBP	6YIL	-8.4
	CAV1	AF-Q03135-F1	-5.7
	PTGS2	5F1A	-9.5
isorhamnetin	CTNNB1	1LUJ	-7
	TP53	6SL6	-6.7
	EGFR	1M17	-8.6
	SRC	1FMK	-8.1
	CDH1	3FF7	-8.4
	HSP90AA1	1BYQ	-7.1
	MDM2	6KZU	-6.6
	EP300	3BIY	-8.5
	STAT3	5AX3	-8.1
	ERBB2	2A91	-8.7
	GSK3B	1O9U	-7.6
	CREBBP	6YIL	-8.3
	CAV1	AF-Q03135-F1	-6
	PTGS2	5F1A	-8.7
eriodictyol	CTNNB1	1LUJ	-6.9
	TP53	6SL6	-6.8

EGFR	1M17	-8.6
SRC	1FMK	-8.5
CDH1	3FF7	-8.3
HSP90AA1	1BYQ	-7.4
MDM2	6KZU	-6.5
EP300	3BIY	-8.7
STAT3	5AX3	-8.5
ERBB2	2A91	-8.5
GSK3B	1O9U	-7.8
CREBBP	6YIL	-8.5
CAV1	AF-Q03135-F1	-6.2
PTGS2	5F1A	-9.6

Figure S1. The blood drug concentration of kaempferol following BTG administration. Kaempferol standard was obtained from MedChemExpress (USA) with a purity of 99.86% (HY-14590). An appropriate amount of kaempferol was added to 75% methanol to prepare a stock solution of 200 μg/mL for the mixed control solution. Upon usage, it was sequentially diluted to obtain concentration gradient solutions. Utilizing the same 50 female SD rats as employed in the *in vivo* experiment, two rats constituted the blank control group, while the remaining 48 rats were divided into treatment groups, each consisting of 6 rats. The treatment groups received oral gavage of BTG at 3.27 g/kg for 3 consecutive days, while the blank control group received an equivalent volume of physiological saline using the same procedure. Prior to the final dosing, the rats were fasted for 12 hours but allowed access to water. Blood samples were collected from the abdominal aorta of the rats at 8 time intervals post administration (10 min, 20 min, 30 min, 1 h, 2 h, 4 h, 8 h, 12 h) after anesthetizing the rats using 1% pentobarbital sodium. The blood samples were centrifuged at 4°C, 3500 rpm for 10 min, and the supernatant was analyzed. Following the UPLC-MS analysis method described earlier, the peak areas of the analyte and internal standard were recorded to calculate the blood drug concentrations at each time point.

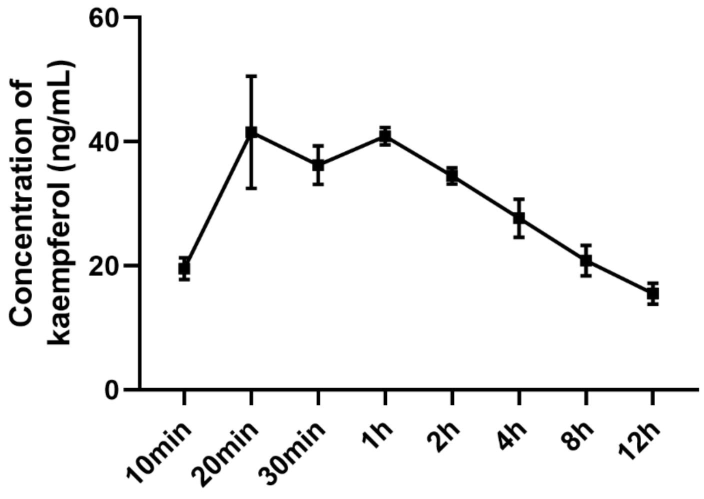

Figure S2. The impact of BTG administration on rat liver and kidney function. The serum levels of (A) ALT, (B) AST, (C) CREA, (D) UA, and (E) UREA in each group of rats were measured using microplate assays. The assay kits were obtained from Nanjing Jiancheng Bioengineering Institute (China).

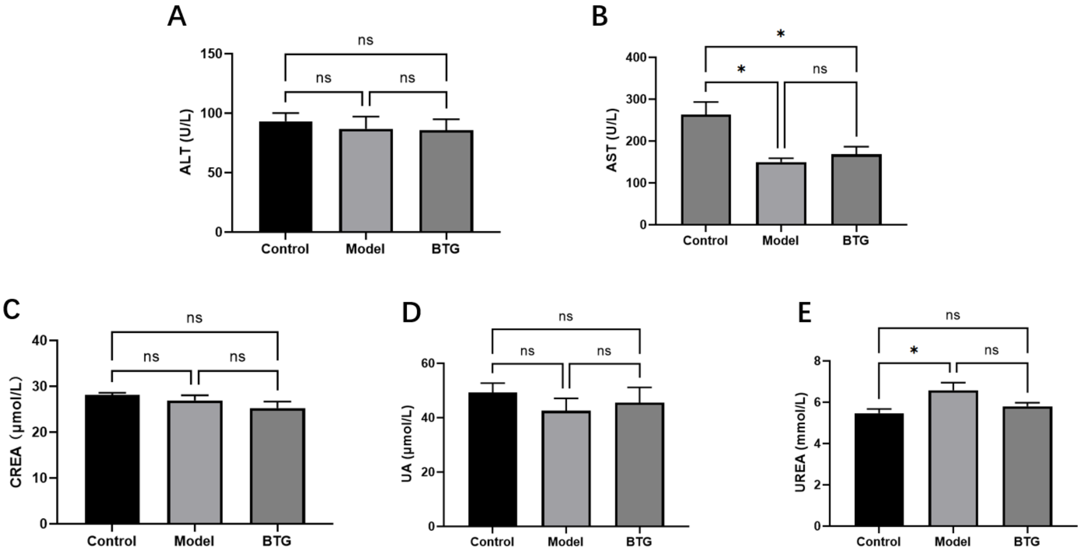

Supplement: Supplementary file 1 — Additional file 1: Table S1. Composition of the BTG. Table S2. Identification of chemical components in BTG. Table S3. Affinity of ingredients with potential targets. Figure S1. The blood drug concentration of kaempferol following BTG administration. Kaempferol standard was obtained from MedChemExpress (USA) with a purity of 99.86% (HY-14590). An appropriate amount of kaempferol was added to 75% methanol to prepare a stock solution of 200 μg/mL for the mixed control solution. Upon usage, it was sequentially diluted to obtain concentration gradient solutions. Utilizing the same 50 female SD rats as employed in the in vivo experiment, two rats constituted the blank control group, while the remaining 48 rats were divided into treatment groups, each consisting of 6 rats. The treatment groups received oral gavage of BTG at 3.27 g/kg for 3 consecutive days, while the blank control group received an equivalent volume of physiological saline using the same procedure. Prior to the final dosing, the rats were fasted for 12 hours but allowed access to water. Blood samples were collected from the abdominal aorta of the rats at 8 time intervals post administration (10 min, 20 min, 30 min, 1 h, 2 h, 4 h, 8 h, 12 h) after anesthetizing the rats using 1% pentobarbital sodium. The blood samples were centrifuged at 4°C, 3500 rpm for 10 min, and the supernatant was analyzed. Following the UPLC-MS analysis method described earlier, the peak areas of the analyte and internal standard were recorded to calculate the blood drug concentrations at each time point. Figure S2. The impact of BTG administration on rat liver and kidney function. The serum levels of (A) ALT, (B) AST, (C) CREA, (D) UA, and (E) UREA in each group of rats were measured using microplate assays. The assay kits were obtained from Nanjing Jiancheng Bioengineering Institute (China). [file 13048_2023_1339_MOESM1_ESM.pdf]
